# The Role of Millimeter-Waves in the Distance Measurement Accuracy of an FMCW Radar Sensor

**DOI:** 10.3390/s19183938

**Published:** 2019-09-12

**Authors:** Akanksha Bhutani, Sören Marahrens, Michael Gehringer, Benjamin Göttel, Mario Pauli, Thomas Zwick

**Affiliations:** 1 Institute of Radio Frequency Engineering and Electronics, Karlsruhe Institute of Technology, Campus South, Engesserstrasse 5, 76131 Karlsruhe, Germany; soeren.marahrens@kit.edu (S.M.); michael.gehringer@student.kit.edu (M.G.); mario.pauli@kit.edu (M.P.); thomas.zwick@kit.edu (T.Z.); 2 Wellenzahl Radar- und Sensortechnik GmbH & Co. KG, Im Vogelsand, 76131 Karlsruhe, Germany; benjamin.goettel@wellenzahl.de

**Keywords:** millimeter-wave, frequency modulated continuous wave, frequency estimation, phase estimation, Cramér Rao lower bound, micrometer-accuracy range measurement

## Abstract

High-accuracy, short-range distance measurement is required in a variety of industrial applications e.g., positioning of robots in a fully automated production process, level measurement of liquids in small containers. An FMCW radar sensor is suitable for this purpose, since many of these applications involve harsh environments. Due to the progress in the field of semiconductor technology, FMCW radar sensors operating in different millimeter-wave frequency bands are available today. An important question in this context, which has not been investigated so far is how does a millimeter-wave frequency band influence the sensor accuracy, when thousands of distance measurements are performed with a sensor. This topic has been dealt with for the first time in this paper. The method used for analyzing the FMCW radar signal combines a frequency- and phase-estimation algorithm. The frequency-estimation algorithm based on the fast Fourier transform and the chirp-z transform provides a coarse estimate of the target distance. Subsequently, the phase-estimation algorithm based on a cross-correlation function provides a fine estimate of the target distance. The novel aspects of this paper are as follows. First, the estimation theory concept of Cramér-Rao lower bound (CRLB) has been used to compare the accuracy of two millimeter-wave FMCW radars operating at 60 GHz and 122 GHz. In this comparison, the measurement parameters (e.g., bandwidth, signal-to-noise ratio) as well as the signal-processing algorithm used for both the radars are the same, thus ensuring an unbiased comparison of the FMCW radars, solely based on the choice of millimeter-wave frequency band. Second, the improvement in distance measurement accuracy obtained after each step of the combined frequency- and phase-estimation algorithm has been experimentally demonstrated for both the radars. A total of 5100 short-range distance measurements are made using the 60 GHz and 122 GHz FMCW radar. The measurement results are analyzed at various stages of the frequency- and phase-estimation algorithm and the measurement error is calculated using a nanometer-precision linear motor. At every stage, the mean error values measured with the 60 GHz and 122 GHz FMCW radars are compared. The final accuracy achieved using both radars is of the order of a few micrometers. The measured standard deviation values of the 60 GHz and 122 GHz FMCW radar have been compared against the CRLB. As predicted by the CRLB, this paper experimentally validates for the first time that the 122 GHz FMCW radar provides a higher repeatability of micrometer-accuracy distance measurements than the 60 GHz FMCW radar.

## 1. Introduction

A frequency modulated continuous wave (FMCW) radar is used to measure distance, speed and angle of one or more remote objects without establishing any physical contact with the objects. The measurement capability of an FMCW radar is by no means affected by poor visibility conditions (e.g., rain, snow, fog and night-darkness) and other harsh environmental conditions (e.g., flying debris and pollution), which gives it a clear edge over optical systems such as infra-red, lidar and cameras. Therefore, FMCW radars are used in a wide variety of applications ranging from consumer applications e.g., automotive and security sector, to industrial applications e.g., tracking and positioning of machines and robots in a fully automated production process, level measurement of liquids in small containers and vibration measurement. Many industrial applications require micrometer-accuracy distance measurements over a short range (i.e., of the order of a few meters). Some of these applications are as follows: Measuring the embossment depth of car body IDs, which are punched into the vehicle frame for theft protection, measuring the thickness of silicon wafers and detecting warpage in these wafers, measuring the curvature of optical lenses, gap measurement in axial piston pumps, thickness measurement of safety glass and so on. All these applications require non-contact distance measurement with an accuracy of a few micrometers. Such an accuracy can be achieved with an FMCW radar, if its intermediate frequency (IF) signal is analyzed using a combined frequency- and phase-estimation algorithm. Both algorithms, when used independently, have a certain limitation. A frequency-estimation algorithm can measure distances unambiguously much longer than half of the wavelength but its accuracy is limited. On the other hand, a phase-estimation algorithm can provide very high distance-measurement accuracy but its unambiguous range is limited to half of the wavelength. Therefore, a combination of both the algorithms is considered suitable for the purpose at hand. In [1], such a method has been described and its implementation in a 24 GHz FMCW radar has been shown. In [2], a high-accuracy 24 GHz FMCW radar is used to detect the position of a piston in a hydraulic cylinder. Further, the influence of FMCW ramp signal parameters, including ramp nonlinearity, phase noise and signal-to-noise ratio (SNR) on the distance measurement accuracy of a 24 GHz FMCW radar is investigated in [3] and the influence of a target’s geometry and position on the accuracy of a broadband 80.5 GHz FMCW radar is shown in [4]. The majority of these and several other papers published in this field are based on FMCW radars operating below 100 GHz. There are a few exceptions such as [5] presents a D-Band FMCW radar with 48 GHz bandwidth, which shows a distance measurement accuracy of less than 1 μm for a target placed at a distance of 65 cm. Recently a 240 GHz FMCW radar with a large bandwidth of 52 GHz has been demonstrated in [6], which shows a compensated distance measurement accuracy of 0.5 μm over an ultra-short measurement range of 27 to 28 cm. It is interesting to note that the progress made in the field of semiconductor technology has made a variety of FMCW radars available to us. In less than a decade, at least in the research area, the legacy 24 GHz FMCW radar has been replaced by FMCW radars operating in the millimeter-wave frequency bands, including 60 GHz, 77 GHz, 122 GHz, etc. Therefore, the question arises as to whether the same trend should be followed in the industry as well. Although increasing the operating frequency of an FMCW radar provides miniaturization benefits, it comes hand in hand with an increasing free space path loss. Another important question to be answered is if the choice of the millimeter-wave frequency band that influences the distance measurement accuracy of an FMCW radar. This problem has been dealt for the first time in this paper. A 60 GHz and a 122 GHz FMCW radar have been used for this investigation. The high accuracy distance measurements have been made over a distance of a few meters, as typically encountered in an industrial scenario. For each radar a total of 5100 continuous distance measurements are made in order to compare their performance and hence draw conclusions regarding their accuracy and repeatability. The continuous distance measurements are made using a nanometer-precision linear motor. The motor also serves as the reference for calculating the distance measurement errors of the radars under test. The performance of the radars is compared as follows. First, the mean values of the distance measurement error sets (each radar under test has a total of 51 error sets and each error set consists of 100 error values) are calculated at multiple stages of the frequency- and phase-estimation algorithm, and compared for both the radars. Second, the standard deviation values of the distance measurement error sets are calculated at the last stage of the frequency- and phase-estimation algorithm. The standard deviation values of the 60 GHz and 122 GHz FMCW radars are experimentally validated against the widely known estimation theory concept of Cramér-Rao lower bound (CRLB). This paper is organized as follows. Section 2 begins with the FMCW radar principle. Next, the frequency- and phase-estimation algorithms used for enhancing the distance measurement accuracy of an FMCW radar are described in detail. Towards the end of this section, the measurement setup conditions, which tend to degrade the accuracy of the frequency- and phase-estimation methods, are described. Besides, the CRLB equations for a frequency- and phase-estimation method are shown, which specify the minimum theoretical limit of the variances in these two methods. Thereafter, the measurement setup, the 60 GHz and 122 GHz FMCW radars used in this investigation are shown. The specifications of the radars under test, the chosen target and the reference linear motor are given. In Section 3, the distance measurement results of both the radars are compared at multiple stages of the frequency- and phase-estimation method and the mean error values measured with both the radars are compared at each stage. The theoretically expected measurement error is also calculated at the first two stages, thus highlighting the influences in a real measurement scenario. The measured standard deviation values of the 60 GHz and 122 GHz FMCW radars are compared and hence an experimental validation of the CRLB is presented. In Section 4, the measurement results are discussed and the most important conclusions drawn from this work are presented.

## 2. Materials and Methods

### 2.1. Conventional FMCW Radar

**Figure 1:**
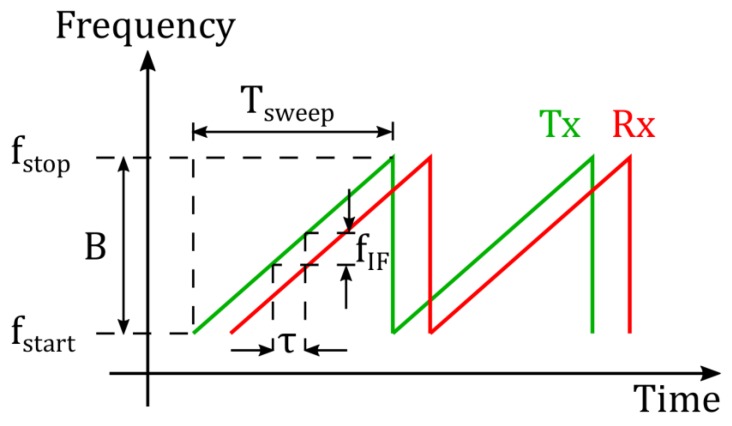
Frequency modulated continuous wave (FMCW) radar principle.

The basic principle of an FMCW radar is shown in Figure 1. A detailed description of this concept is given in [7]. The transmitted (Tx) signal is a linear frequency modulated wave, which periodically sweeps over a bandwidth of $B=\left(f_{\mathrm{stop}}-f_{\mathrm{start}}\right)$ within a time period of $T_{\mathrm{sweep}}$. The received (Rx) signal is a time-delayed version of the Tx signal, where the time-delay $\left(\tau =2R/c_{0}\right)$ corresponds to the time taken by the radar signal in travelling to and from a target (R and $c_{0}$ denote the target distance and the speed of light, respectively). The difference of the Tx and Rx signals, known as the intermediate frequency (IF) signal, is used to measure the target distance as shown in Equation (1).

(1) $$f_{\mathrm{IF}}=\;\frac{2RB}{c_{0}T_{\mathrm{sweep}}}.$$

The parameters B, $T_{\mathrm{sweep}}$ and $c_{0}$ are fixed for a given radar. Therefore, the distance measurement accuracy of an FMCW radar is limited by the accuracy with which the value of $f_{\mathrm{IF}}$ is estimated. In a typical FMCW radar, the value of $f_{\mathrm{IF}}$ is estimated in the following manner. A finite number of IF signal samples (in time domain) are captured by an analog-to-digital converter (ADC). These samples are windowed, which reduces the side lobe level at the cost of an increase in the target peak width. In other words, windowing reduces the effect of multiple spectral peaks at the cost of a reduced resolution [8]. Thereafter, the samples are transformed into the frequency domain by using the fast Fourier transform (FFT). The peak of the FFT spectrum provides the $f_{\mathrm{IF}}$ value. The limitations in this approach are as follows.

- The estimation accuracy of $f_{\mathrm{IF}}$ is limited by the bin width of the FFT spectrum (FFT bin width is the frequency gap between two consecutive spectral lines in the FFT spectrum). The FFT bin width is given by $F_{s}/N,$ where $F_{s}$ and N denote the sampling frequency and the number of samples, respectively.
- Zero padding is used to interpolate the FFT spectrum and hence increase its frequency resolution. However, zero padding leads to a massive increase in the computational complexity.
- If two or more targets are located so close to each other such that their corresponding peaks in the frequency domain lie within the 3 dB width of the chosen window function, then no amount of zero padding can resolve the target peaks.

### 2.2. Enhancing the Distance Measurement Accuracy of an FMCW Radar

The distance measurement accuracy of an FMCW radar is enhanced by combining a frequency- and phase-estimation method for analyzing the IF signal samples. Two variations of this method are shown in [1,9]. These methods are explained in the following sections.

#### 2.2.1. Frequency-Estimation Algorithm

**Figure 2:**
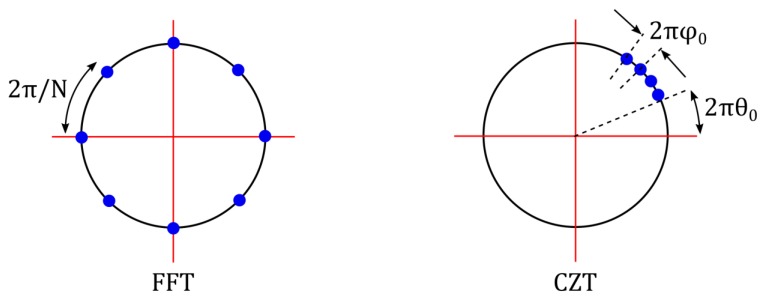
Z-plane unit circle representation of the fast Fourier transform (FFT) and the chirp z transform (CZT).

The FFT is a faster version of the discrete Fourier transform (DFT) shown by Equation (2), which reduces the number of complex multiplications and additions from $N^{2}$ to $N\log_{2}N$ (N denotes the number of FFT or DFT points), provided N is a power of 2. In addition, the FFT is calculated at equidistant points along the *z*-plane unit circle (as shown in Figure 2), which explains why it is impractical to achieve an extremely high frequency resolution and hence a high distance measurement accuracy using the FFT alone.

(2) $$X_{k}=\sum_{n=0}^{N-1}x_{n}e^{\left(-j2\pi k/N\right)}\;\;\;\;\;\;\;k=0,1,\ldots ,N-1.$$

This limitation of the FFT is overcome by using the chirp z transform (CZT). In the mathematical expression of the CZT (see Equation (3)), $A=A_{0}e^{j2\pi \theta_{0}}$ and $W=W_{0}e^{j2\pi \varphi_{0}}$ are arbitrary complex numbers. Moreover, Equation (3) becomes the same as Equation (2), if A=1, $W=e^{\left(-j2\pi /N\right)}$ and M=N. Therefore, it indicates that the CZT is a generalized form of the DFT or the FFT. The main advantage of the CZT lies in its calculation flexibility as shown by the following points. First, the CZT is calculated along an arbitrary z-plane contour, which starts at a random point A on the z-plane. Second, unlike the FFT, the contour over which the CZT is calculated is a small section of the unit circle on the z-plane, which spirals in or out with respect to the origin depending on the value of W. Third, the angular spacing between the consecutive points is an arbitrary value given by $2\pi \varphi_{0}$. Finally, in contrast with the FFT, the number of time- and frequency-domain samples (i.e., N and M, respectively) need not be equal in the CZT [10,11,12].

(3) $$X_{k}=\sum_{n=0}^{N-1}x_{n}A^{-n}W^{nk}\;\;\;\;\;\;\;\;k=0,1,\ldots ,M-1.$$

A z-plane representation of the CZT with A=1 is shown in Figure 2. When the CZT equation (i.e., Equation (3)) is implemented as it is, it requires *NM* complex multiplications and additions. However, if *nk* in Equation (3) is substituted by the expression $\left(n^{2}+k^{2}-{\left(k-n\right)}^{2}\right)/2$ as proposed by Bluestein [13], then the number of computations required in implementing the CZT is reduced to nearly $\left(N+M\right)\log_{2}\left(N+M\right)$ [10,11]. Consequently, the CZT is capable of estimating the IF frequency with a much higher accuracy than the FFT and this high accuracy is achieved at the expense of a moderately higher computational effort than the FFT. In other words, the FFT is suitable for analyzing a broadband frequency range; hence suitable for providing a coarse estimation of the IF frequency. On the other hand, the CZT is suitable for analyzing a narrowband frequency range; hence suitable for providing a fine estimation of the IF frequency. In this work, the FFT was used in conjunction with the CZT in order to optimize the frequency-estimation algorithm. The main steps of this algorithm are described below.

1. The time-domain samples captured by the ADC are used to calculate the FFT over a frequency span of $\left[-F_{s}/2,\;F_{s}/2\right]$. The zero padding factor is kept as one; hence the number of FFT points (N) is the same as the number of ADC samples. The frequency corresponding to the peak amplitude of the FFT spectrum is termed as the coarse frequency estimate $\left(f_{\mathrm{FFT}}\right)$. In an ideal scenario, when $f_{\mathrm{FFT}}$ is substituted in Equation (1), it provides an absolutely accurate value of the target distance (R), provided $f_{\mathrm{FFT}}$ is an integral multiple of the FFT bin width (i.e., $F_{s}/N$). In every other case, $f_{\mathrm{FFT}}$ leads to an error in the distance measurement. This error has a maximum limit of one-half of the FFT bin width on either side of the FFT spectral line corresponding to $f_{\mathrm{FFT}}$; hence the ideal frequency estimate is given by Equation (4).
  (4) $$f_{\mathrm{ideal}}=f_{\mathrm{FFT}}\pm {\frac{F_{s}}{2N}}_{\;}.$$
2. The CZT is calculated for the ADC samples over a narrowband frequency range, whose lower and upper limits are given by Equations (5) and (6), respectively.
  (5) $$f_{\mathrm{lower}\_\mathrm{CZT}}=f_{\mathrm{FFT}}-{\frac{F_{s}}{N}}_{\;}.$$
  (6) $$f_{\mathrm{upper}\_\mathrm{CZT}}=f_{\mathrm{FFT}}+{\frac{F_{s}}{N}}_{\;}.$$
  The CZT frequency interval is kept slightly larger than required (i.e., two FFT bins instead of one are used) due to practical reasons. The number of points at which the CZT is calculated (M) is same as the ADC sample length (N). The values of complex numbers A and W, which define the z-plane contour used for calculating the CZT, are given by Equations (7) and (8), respectively.
  (7) $$A=\exp\left(\frac{j2\pi f_{\mathrm{lower}\_\mathrm{CZT}}}{F_{s}}\right).$$
  (8) $$W=\exp{\left(\frac{-j2\pi {\left(f_{\mathrm{upper}\_\mathrm{CZT}}-f_{\mathrm{lower}\_\mathrm{CZT}}\right)}_{\;}}{MF_{s}}\right)}^{\;}.$$
  The frequency corresponding to the peak value of the CZT spectrum provides a finer frequency estimate ($f_{\mathrm{CZT}1}$), whose accuracy is better than the accuracy of $f_{\mathrm{FFT}}$ by a factor of 1/M. Consequently, the value of $f_{\mathrm{ideal}}$ (i.e., the frequency corresponding to the ideal target distance) after calculating the CZT is given by Equation (9)
  (9) $$f_{\mathrm{ideal}}=f_{\mathrm{CZT}1}\pm {\frac{F_{s}}{2NM}}_{\;}.$$
3. The CZT is calculated a second time to get an even finer estimate of the IF frequency i.e., $f_{\mathrm{CZT}2}$. In an ideal scenario, which contains a single target peak in the frequency spectrum, the accuracy of the estimated IF frequency ($f_{\mathrm{CZTk}}$) improves by a factor of $1/M^{k}$ after the $k^{\mathrm{th}}$ iteration of the CZT as shown by Equation (10). However, in a real scenario, the target peak is influenced by the presence of effects such as other spectral peaks arising from surrounding objects, various types of noise and windowing, which restrict the highest achievable accuracy of the IF frequency estimate. In this work, it is shown that two CZT iterations are found to be sufficient to achieve this accuracy limit of the IF frequency estimate.
  (10) $$f_{\mathrm{ideal}}=f_{\mathrm{CZTk}}\pm {\frac{F_{s}}{2NM^{k}}}_{\;}.$$

**Figure 3:**
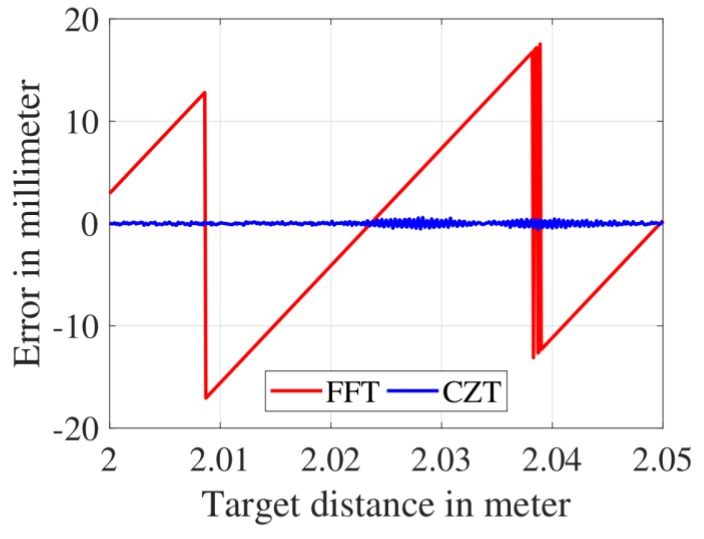
Comparison of the distance measurement error with the FFT and CZT.

The advantage of the CZT over the FFT can be clearly seen from the comparison shown in Figure 3. In this plot, the distance measurement accuracy of an FMCW radar after performing the FFT versus the CZT (i.e., the first and second step of the abovementioned algorithm, respectively) is shown. The sawtooth pattern observed in the FFT distance measurement error can be explained in the following manner. If the target distance measured by an FMCW radar is such that the corresponding IF frequency ($f_{\mathrm{IF}}$) is an exact multiple of the FFT bin width (i.e., the $f_{\mathrm{IF}}$ coincides with one of the discrete spectral lines of the FFT spectrum), then the radar is able to determine the $f_{\mathrm{IF}}$ value with 100% accuracy and the distance measurement error is zero in this case. As the target distance changes and the corresponding $f_{\mathrm{IF}}$ does not coincide with any of the discrete spectral lines of the FFT spectrum (i.e., $f_{\mathrm{IF}}$ is not an integral multiple of the FFT bin width), it leads to an error in estimating the $f_{\mathrm{IF}}$ and hence the target distance. In this case, the smallest IF frequency, which can be resolved by the FFT is given by $\Delta f_{\mathrm{IF}}=F_{s}/N=3.9\;\mathrm{kHz}$ ($F_{s}=$ 2 MHz, N= 512). The FMCW radar parameters used in this case were: B= 5 GHz and $T_{\mathrm{sweep}}=$ 256 μs. On substituting the $\Delta f_{\mathrm{IF}}$ and the radar parameters in Equation (1), a target distance of 30 mm was obtained, which is the same as the period of the FFT curve seen in Figure 3. Moreover, the FFT distance measurement error observed in Figure 3 varies between ±15 mm. The same principle applies to the CZT as well. The CZT bin width was several orders of magnitude smaller than the FFT and therefore the CZT distance measurement error was nearly 15 times lower than the FFT distance measurement error.

##### 2.2.2. Phase-Estimation Algorithm

The importance of phase estimation in enhancing the distance measurement accuracy of an FMCW radar can be understood from the following example. In this example, a 60 GHz FMCW radar with the following parameters was taken into consideration: B= 5 GHz and $T_{\mathrm{sweep}}=$ 256 μs. If the distance of a target changes by a small amount of ΔR= 100 μm, the corresponding change in the IF frequency was calculated as $\Delta f_{\mathrm{IF}}=$ 13 Hz (using Equation (1)). If the IF frequency is estimated using the FFT alone ($F_{s}=$ 2 MHz, N= 512), then the FFT bin width ($F_{s}/N=$ 3.9 kHz) is insufficient to resolve such a small change in the IF frequency. Subsequently, if the CZT (M=512) is used as described in step 2 of the frequency-estimation algorithm in Section 2.2.1, the CZT bin width is calculated as $\left(f_{\mathrm{upper}\_\mathrm{CZT}}-f_{\mathrm{lower}\_\mathrm{CZT}}\right)/M=2F_{s}/NM=$ 15.2 Hz, which is again higher than the desired $\Delta f_{\mathrm{IF}}$. Hence, at least two CZT iterations are necessary in this case. Moreover, the time interval associated with the $\Delta f_{\mathrm{IF}}$ value is approximately $0.003T_{\mathrm{sweep}}=$ 768 ns. On the other hand, the phase change associated with ΔR is calculated using Equation (11).

(11) $$\Delta \varphi_{\mathrm{IF}}=\frac{4\pi \Delta R}{\lambda_{\mathrm{start}}}.$$

$\lambda_{\mathrm{start}}$ denotes the wavelength corresponding to the start frequency of the FMCW ramp. The start frequency of the FMCW ramp is given by 60 GHz−B/2= 57.5 GHz and its corresponding wavelength is $\lambda_{\mathrm{start}}=5.21\;\mathrm{mm}$. The corresponding phase change was calculated as $\Delta \varphi_{\mathrm{IF}}≅14{}^{\circ}$. On comparing the $\Delta f_{\mathrm{IF}}$ and $\Delta \varphi_{\mathrm{IF}}$ values, we saw that the $\Delta f_{\mathrm{IF}}$ was associated with a very small time interval of the FMCW ramp cycle, whereas the $\Delta \varphi_{\mathrm{IF}}$ was a significant fraction of the unambiguous phase range [−π, π]. Therefore, it was observed that the phase value was more sensitive to a small change in the target distance and hence it was relatively easier to discern. The only limitation in this case was that the unambiguous range was much smaller (i.e., one-half of the wavelength) than in the frequency estimation case. Considering these points, the phase estimation algorithm consists of the following steps.

**Figure 4:**
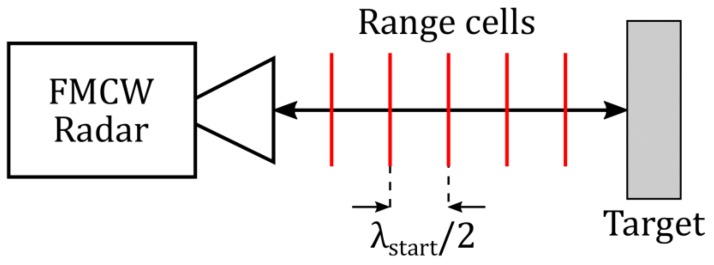
Range cell concept.

1. The radar-to-target distance is divided into n number of unambiguous range cells as shown in Figure 4. The length of each range cell is *λ*_start_/2. As described in Section 2.2.1, the frequency estimation algorithm provides an IF frequency estimate termed $f_{\mathrm{CZT}2}$ after executing the FFT and two iterations of the CZT. On substituting $f_{\mathrm{IF}}=f_{\mathrm{CZT}2}$ in Equation (1), a coarse estimate of the target range ($R_{\mathrm{coarse}}$) is obtained, which indicates the $n^{th}$ range cell in which the target is located, as shown by Equation (12).
  (12) $$R_{\mathrm{coarse}}=\frac{n\lambda_{\mathrm{start}}}{2}.$$
2. The initial phase of the IF signal $(\varphi_{\mathrm{IF}}$) is equal to the phase difference of the Tx and Rx FMCW chirps (note that the term ‘chirp’ is used instead of ‘ramp’, since the phase estimation algorithm is implemented in the time domain) at that instance of time. Therefore, the information regarding the time delay between the Tx and Rx chirps is stored in the $\varphi_{\mathrm{IF}}$ value. Hence, within the $n^{th}$ range cell, a fine estimate of the target distance $\left(R_{\mathrm{fine}}\right)$ is obtained from $\varphi_{\mathrm{IF}}$ by using Equation (13). The final estimation of the target distance is given by Equation (14) [1].
  (13) $$R_{\mathrm{fine}}=\frac{\;\varphi_{\mathrm{IF}}\;\lambda_{\mathrm{start}}}{4\pi }.$$
  (14) $$R_{\mathrm{abs}}=R_{\mathrm{coarse}}+R_{\mathrm{fine}}.$$
3. The value of $\varphi_{\mathrm{IF}\;}$ is obtained by executing a cross-correlation function between the IF signal captured by the ADC and a reference complex harmonic signal, which is formed using $f_{\mathrm{CZT}2}$ (obtained from frequencyestimation algorithm). These two signals are shown by Equations (15) and (16), respectively, and their cross-correlation function is shown by Equation (17).
  (15) $$x\left[t\right]=A_{\mathrm{IF}}\cos\left(2\pi f_{\mathrm{IF}}^{\prime }t+\varphi_{\mathrm{IF}}\right).$$
  (16) $$y\left[t\right]=\exp{\left(-j2\pi f_{\mathrm{CZT}2}^{\prime }t\right)}_{\;}.$$
  (17) $$C_{\mathrm{xy}}\left[t\right]=\sum_{t=0}^{N-1}x\left[t\right]y\left[t\right].$$
  $f_{\mathrm{IF}}^{\prime }$ and $f_{\mathrm{CZT}2}^{\prime }$ denote normalized values of $f_{\mathrm{IF}}$ and $f_{\mathrm{CZT}2}$ respectively, with respect to $F_{S}$. The noise embedded in the signal x[t] being uncorrelated gets suppressed during cross-correlation. Further, the cross-correlation results in a sinc function, whose argument provides the value of $\varphi_{\mathrm{IF}\;}.$ Alternatively, $\varphi_{\mathrm{IF}}$ can also be determined from the peak of the frequency spectrum calculated using the CZT. This alternative method has been used in [1].

##### 2.2.3. Limitations of the Frequency- and Phase-Estimation Algorithm

The frequency- and phase-estimation algorithms described above have certain limitations, which were taken into consideration, while performing the high-accuracy distance measurements shown in this work. In the frequency-estimation algorithm, the accuracy of the CZT iterations is dependent on the initial value of the IF frequency calculated using the FFT (i.e., $f_{\mathrm{FFT}}$). If the target peak in the FFT spectrum is shifted from its actual position, then even a higher number of CZT iterations can not improve the accuracy of the estimated IF frequency. The shifting of the target peak may occur due to the following reasons. First, if the target is located near the end of the unambiguous range (The unambiguous range of a radar is defined in several radar books e.g., [14]). In this case, the normalized IF frequency is given by $f_{\mathrm{IF}}^{\prime }≅0.5$. The periodic nature of the FFT leads to spectral leakage, which shifts the target peak. Second, if the target is located very close to the radar, the value of $f_{\mathrm{IF}}^{\prime }$ is very low ($f_{\mathrm{IF}}^{\prime }≅0$). In this case, the presence of a DC peak (a DC peak always exists in a real bistatic or monostatic radar) distorts the target peak. Besides, even in an ideal scenario, the symmetrical nature of the FFT causes spectral leakage from the negative portion of the frequency spectrum thus shifting the target peak. Third, one or more objects present in the vicinity of the target also distort the target peak. In the phase-estimation algorithm, a high accuracy is achieved, if and only if the target range accuracy achieved after implementing the frequency estimation algorithm is less than $\lambda_{\mathrm{start}}/4$, as shown in Equation (18) [1]. If this condition fails, then the value of $\varphi_{\mathrm{IF}}$ may lie close to 0° (i.e., start of a range cell) or 180° (i.e., end of a range cell), which can shift the final estimation of the target distance ($R_{\mathrm{abs}}$) to an incorrect range cell.

(18) $$\left|R_{\mathrm{ideal}}-R_{\mathrm{coarse}}\right|\leq \;\frac{\lambda_{\mathrm{start}}}{4}.$$

Up to this point, the target range accuracy for a single FMCW radar measurement was discussed. However, if an FMCW radar is used in an industrial or consumer application, then hundreds of thousands of target range measurements are made and hence the accuracy is expressed in terms of a mean and a standard deviation (σ) value. The standard deviation is the square root of variance ($\sigma^{2}$) and the minimum possible value of variance is limited by a theoretical limit, known as the Cramér Rao lower bound (CRLB). As per CRLB, if an FMCW radar is used to repeatedly measure a target distance based on a frequency-estimation method, then the variance of this set of measured values is bound by the expression shown in Equation (19). Additionally, if a phase-estimation method is used for this purpose, then the CRLB of the variance is given by Equation (20) [15].

(19) $$\sigma_{\mathrm{freq}}^{2}\geq \frac{3c_{0}^{2}}{4\pi^{2}\eta NB^{2}}.$$

(20) $$\sigma_{\mathrm{phase}}^{2}\geq \frac{c_{0}^{2}}{4\pi^{2}\eta Nf_{\mathrm{start}}^{2}}.$$

The variance of both frequency- and phase-estimation method decreases with an increase in the SNR denoted by η and the number of IF signal samples denoted by N. Additionally, an important difference between these two bounds is that the variance in a frequency-estimation method decreases with an increase in radar bandwidth (B), whereas the variance of a phase-estimation method decreases with an increase in the start frequency of an FMCW radar $(f_{\mathrm{start}}$). Since a combined frequency- and phase-estimation algorithm is used in this work, the influence of $f_{\mathrm{start}}$ on the variance of a set of estimated target distances is investigated by considering FMCW radars operating in two different frequency bands.

#### 2.3. Measurement Setup

**Figure 5:**
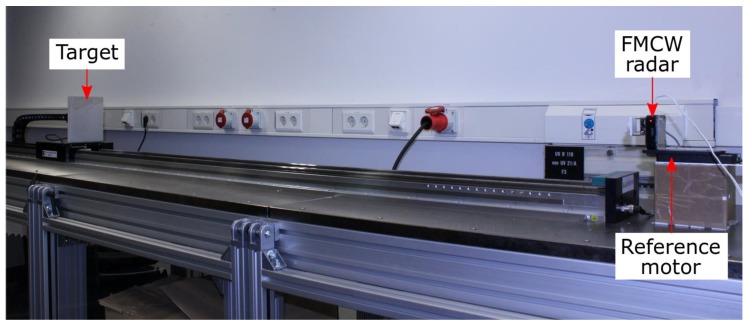
High-accuracy distance measurement setup.

**Figure 6:**
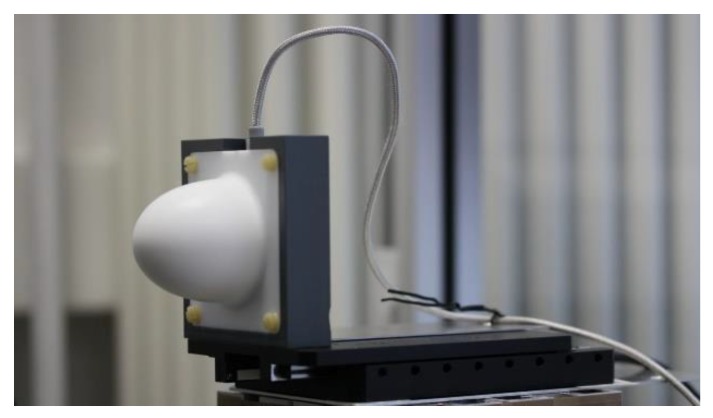
60 GHz FMCW radar.

**Figure 7:**
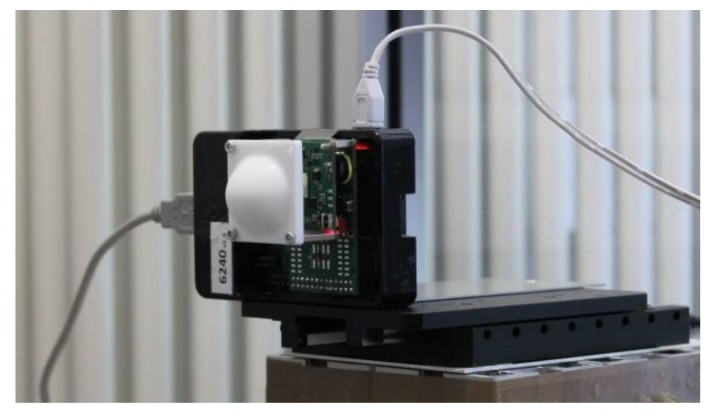
122 GHz FMCW radar.

The high-accuracy distance measurement setup is shown in Figure 5. The FMCW radars under test include a 60 GHz and a 122 GHz FMCW radar, shown in Figure 6 and Figure 7, respectively. The 60 GHz FMCW radar is from Infineon Technologies AG (BGT60TR24B). The device employs an embedded wafer level ball grid array package. The transceiver chip architecture and the packaging concept used in this radar are similar to those shown in [16]. The 122 GHz FMCW radar is a low temperature co-fired ceramic (LTCC) based system-in-package, which consists of a transceiver chip from Silicon Radar GmbH and a baseband module from Wellenzahl Radar- und Sensortechnik GmbH and Co. KG. This radar has been demonstrated in [17]. The antenna size of the 122 GHz FMCW radar is nearly one-fourth of that of the 60 GHz FMCW radar. Both radars are bistatic in nature, i.e., they have separate Tx and Rx antennas. Since the size of an antenna gets smaller as the frequency increases, the distance between the Tx and Rx antenna phase centers in the 122 GHz FMCW radar is much smaller than the distance in the 60 GHz FMCW radar. Therefore, if a lens is mounted on both the radars, the lens focal point is aligned closer to the Tx and Rx antenna phase centers in the 122 GHz FMCW radar [18]. In this work, the radars were integrated with a special lens, which suitably enhanced the Tx and Rx antenna gain in both radars. Both the lenses were made of polytetrafluoroethylene (PTFE). The focal length and diameter of the lens used with the 60 GHz FMCW radar were 5 mm and 50 mm, respectively. The focal length and diameter of the lens used with the 122 GHz FMCW radar were 15 mm and 30 mm, respectively. Both lenses provided a gain of 25 dBi. The key parameters of both radars are given in Table 1. The following parameters: *B*, $T_{\mathrm{sweep}}$ and Tx power ($P_{\mathrm{Tx}}$) of these FMCW radars were adjusted in a manner so that a similar value of η was achieved for both radars. The expression for η is shown by Equation (21) [14].

(21) $$\eta =\frac{P_{\mathrm{Tx}}^{\;}G^{2}\sigma_{\mathrm{RCS}}^{\;}\lambda^{2}}{{\left(4\pi \right)}^{3}R^{4}kTBF}.$$

In this equation, $\sigma_{\mathrm{RCS}}^{\;},\;\lambda ,\;k,\;T$ and F denote the radar cross section (RCS) of the target, wavelength of operation, Boltzmann constant, absolute temperature and noise factor, respectively. The target used in the measurement setup was a flat metal plate with 15 cm side length. The maximum RCS of a flat plate was given by $\sigma_{\mathrm{RCS}}^{\;}=4\pi A^{2}/\lambda^{2}$, where A is the flat plate area. The flat plate was kept at a distance of R= 2.5 m from the radar in each case. The choice of this target distance is explained on the basis of the following points.

- As the target distance increases, the time delay between the Tx and Rx signal (τ) becomes a significant fraction of the sweep time ($T_{\mathrm{sweep}}^{\;}$). In other words, the overlap between the Tx and Rx signal decreases (see Figure 1) and hence lesser number of samples are available for estimating the target distance.
- The radar SNR (η) decreases drastically with an increase in the target distance (R), since $\eta \propto 1/R^{4}$ as shown in Equation (21). This in turn degrades the distance measurement accuracy as shown by the CRLB frequency and phase equations of Equations (19) and (20), respectively.
- In order to perform distance measurements with an accuracy of a few micrometers, an extremely stable measurement setup, free from the influence of mechanical vibrations, is required. The measurement setup shown in Figure 5 provides a maximum target distance of around 2.5 m.

A single target scenario was ensured by placing absorbers around the setup (absorbers are not shown in Figure 1), thus avoiding multipath reflections. Note that the chosen target distance results in a normalized IF frequency, which did not lie in the vicinity of 0.5 or 0 for both radars; hence the spectral leakage problems, which arise due to the FFT periodicity or due to the presence of a DC peak were avoided. One by one, the FMCW radars were mounted on an ultrasonic piezomotor (M-683.2U4 PI) with a positioning accuracy of 100 nm [19]. The motor was used to move the radar over a range of 5 mm in steps of 100 μm each. As a result, the target distance varied from 2.500 m to 2.505 m. At each motor position (total 51), the FMCW radar repeated the distance measurement 100 times thus resulting in a total of 5100 measurements for each radar. The mean and standard deviation value of the distance measurement error was calculated at each motor position by using a set of 100 continuous radar measurements.

## 3. Results

**Figure 8:**
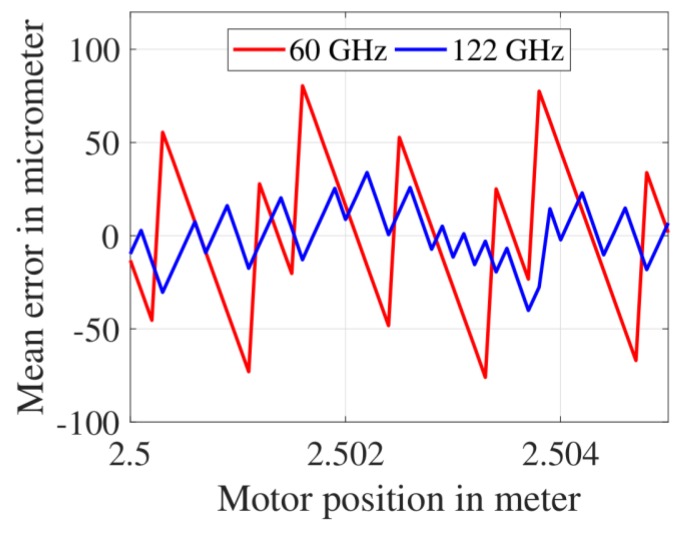
Mean distance measurement error after the first CZT iteration.

**Figure 9:**
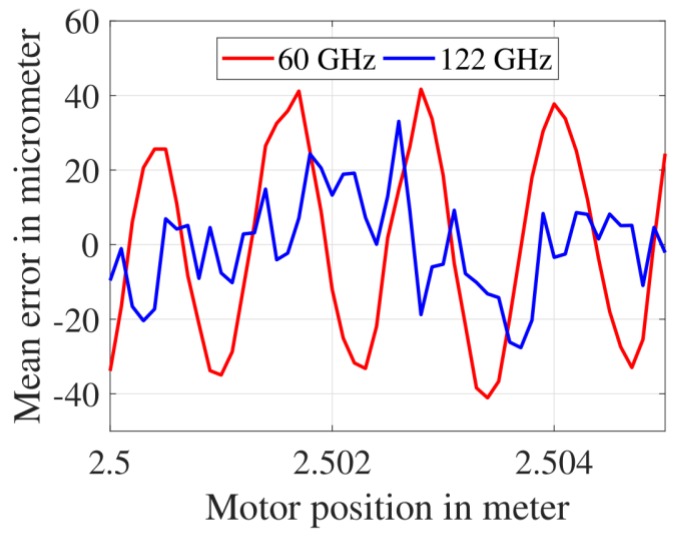
Mean distance measurement error after the second CZT iteration.

**Figure 10:**
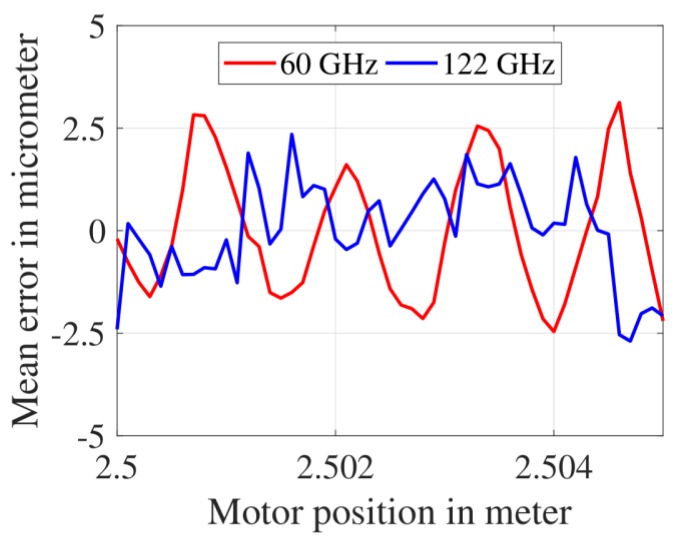
Mean distance measurement error after the combined frequency- and phase-estimation.

**Figure 11:**
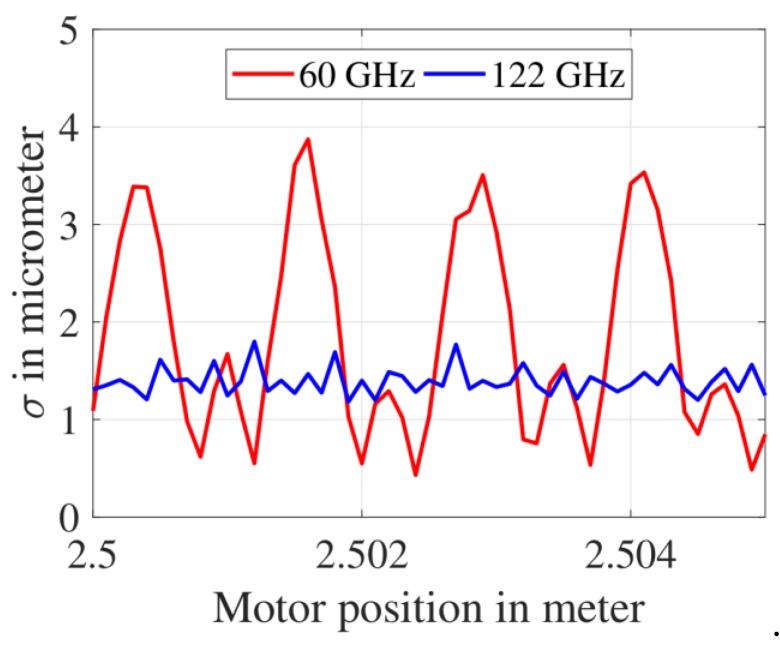
Standard deviation of the final distance measurement errors.

The 60 GHz and 122 GHz FMCW radars were used to perform the high-accuracy distance measurements as described in the previous section. In the measured frequency spectrum of both the radars, the target peak was observed to be around 50 dB higher than the noise and interference level. In other words, the measured SNR as well as the measured signal-to-noise-and-distortion ratio (SNDR) of both the radars was around 50 dB. It should be noted that the effects of baseband circuitry (e.g., ADC quantization error) were included in this measured SNR value, whereas the theoretical radar SNR shown in Equation (21) takes only thermal noise into consideration. The linearity of an FMCW ramp is also a critical factor, which influences the distance measurement accuracy of an FMCW radar. Nonlinearities in an FMCW ramp are divided into two categories. First, random deviations, which occur due to phase noise. These deviations increase the noise floor of the IF signal spectrum and hence degrade the radar SNR. Second, periodic deviations, which occur due to spurious effects arising from e.g., switching in digital circuits and transient response of the phase locked loop. The periodic deviations introduce one or more fixed spurious frequencies in the IF signal spectrum and hence degrade the SNDR. The measured SNR and SNDR of both 60 GHz and 122 GHz FMCW radars was relatively high (50 dB) and hence the FMCW ramp nonlinearities were practically negligible in this scenario. In addition, the frequency-estimation method is followed by the phase-estimation method (i.e., a time-domain correlation method), which further improves the range estimation accuracy [3]. The radar measurement results shown in Figure 8, Figure 9, Figure 10 and Figure 11 are discussed below.

### 3.1. Mean Distance Measurement Error after the First CZT Iteration

The mean value of distance measurement error set obtained after implementing the FFT, followed by the first CZT iteration is shown in Figure 8. At each motor position (from 2.500 m to 2.505 m), the mean error was calculated using 100 FMCW radar measurements. For the 60 GHz FMCW radar, the mean error varied between −76 μm and + 81 μm. For the 122 GHz FMCW radar, the mean error varied between −40 μm and + 34 μm. The span of mean error measured using the 122 GHz FMCW radar was nearly half of that measured using the 60 GHz FMCW radar. The mean error values for the 60 GHz and the 122 GHz FMCW radar could be theoretically verified in the following way. The first CZT iteration estimated the IF frequency with a resolution of $\Delta f_{\mathrm{IF}}=2F_{s}/NM$ (i.e., bin width of the first CZT). This value was calculated as 15.2 Hz for the 60 GHz FMCW radar ($F_{s}=$ 2 MHz, N=M= 512) and 2.4 Hz for the 122 GHz FMCW radar ($F_{s}=$ 5 MHz, N=M= 2048. The values of $F_{s},\;N$ and M are dependent on the value of $T_{\mathrm{sweep}}$, which is shown in Table 1). These two $\Delta f_{\mathrm{IF}}$ values were substituted in Equation (1); hence the theoretically expected distance measurement error of the 60 GHz and 122 GHz FMCW radar was calculated as 116.7 μm and 27.4 μm, respectively.

### 3.2. Mean Distance Measurement Error After the Second CZT Iteration

The mean values of distance measurement error set obtained after implementing the second CZT iteration is shown in Figure 9. In comparison with the previous case, an improvement in terms of the mean error was observed for both the 60 GHz and 122 GHz radars. In addition, it confirmed that the CZT is suitable for narrowband frequency analysis and it provides a finer IF frequency estimate, which in turn provides a finer estimate of the target distance. For the 60 GHz FMCW radar, the mean error varied between −41 μm and + 42 μm. For the 122 GHz FMCW radar, the mean error varied between −27 μm and + 33 μm. Theoretically, the IF frequency resolution after the second CZT iteration, given by $\Delta f_{\mathrm{IF}}=2F_{s}/NM^{2}$, was calculated as 30 mHz and 1.2 mHz for the 60 GHz and the 122 GHz FMCW radar, respectively. The corresponding theoretically expected distance measurement error was calculated as 0.2 μm and 13.8 nm for the 60 GHz and the 122 GHz FMCW radar, respectively. The theoretical error values are hundreds of magnitude smaller than the corresponding measured mean error values. Since the frequency spectrum bin width is of the order of mHz, even a slight distortion of the target peak caused by the effects of a real measurement scenario, is sufficient to induce a distance measurement error of tens of micrometers. Therefore, in this case, successive CZT iterations could not improve the distance measurement accuracy of an FMCW radar.

### 3.3. Mean Distance Measurement Error after Combined Frequency- and Phase-Estimation

The mean distance measurement error achieved with the frequency-estimation method was much lower than the $\lambda_{\mathrm{start}}/4$ value for both radars (i.e., ±40 μm for 60 GHz FMCW radar with $\lambda_{\mathrm{start}}/4=1.3\;\mathrm{mm}$ and ±30 μm for 122 GHz FMCW radar with $\lambda_{\mathrm{start}}/4=625\;\mu m$). Therefore, the phase-estimation method was subsequently implemented in order to further reduce the error for both 60 GHz and 122 GHz FMCW radars. The mean error achieved after implementing the frequency-estimation method (consisting of an FFT and two CZT iterations) and the phase-estimation method is shown in Figure 10. For the 60 GHz FMCW radar, the mean range error varies between −2.5 μm and +3.2 μm and for the 122 GHz FMCW radar, the mean error varied between −2.6 μm and +2.3 μm. The span over which the mean error varied was nearly the same for both FMCW radars.

### 3.4. Standard Deviation of Distance Measurement Error after Combined Frequency- and Phase-Estimation

The standard deviation of the distance measurement error set obtained after implementing the combined frequency- and phase-estimation methods is shown in Figure 11. The maximum standard deviation observed for the 60 GHz and 122 GHz FMCW radar were 3.9 μm and 1.8 μm, respectively. Therefore, the maximum standard deviation (σ) value of the 60 GHz FMCW radar was 2.17 times higher than the 122 GHz FMCW radar; hence the variance $\left(\sigma^{2}\right)$ of the 60 GHz FMCW radar was 4.69 times higher than the 122 GHz FMCW radar. Note that for a phase estimation method, the CRLB of variance is inversely proportional to the square of the start frequency (i.e., $\sigma_{\mathrm{phase}}^{2}\propto 1/f_{\mathrm{start}}^{2}$ from Equation (19)). Further, as per Equation (19), $\sigma_{\mathrm{phase}}^{2}$ also shows a dependence on 1/ηN. However, the value of this product was found to be similar for the 60 GHz and 122 GHz FMCW radar; hence the factor of 1/ηN was ignored in calculating the ratio of variances. Therefore, the ratio of variance of the 60 GHz and the 122 GHz FMCW radars was calculated as shown in Equation (22). The theoretical value is in close agreement with the measured value of 4.69. A summary of the 60 GHz and 122 GHz FMCW radar measurements is shown in Table 2. For simplicity, the absolute maximum value of the mean distance measurement error is shown in the first three columns.

(22) $$\frac{\sigma_{\mathrm{phase}}^{2}\left(60\;\mathrm{GHz}\right)}{\sigma_{\mathrm{phase}}^{2}\left(122\;\mathrm{GHz}\right)}=\frac{{\left(120\;\mathrm{GHz}\right)}^{2}}{{\left(57.5\;\mathrm{GHz}\right)}^{2}}=4.36.$$

## 4. Discussion

In this work, high-accuracy, short-range distance measurements were performed using a 60 GHz and 122 GHz FMCW radar. An accuracy of a few micrometers was achieved in both cases by using a combined frequency- and phase-estimation method. In addition, the CRLB for phase estimation was experimentally validated. The following conclusions are drawn on the basis of the measurement results.

- In terms of frequency estimation, the implementation of the FFT, followed by the two CZT iterations is sufficient to achieve a distance measurement error of tens of micrometers, provided standard FMCW radar parameters (e.g., bandwidth, sweep time and Tx power) and an optimum measurement scenario (e.g., single target and short range), as shown in this work are used. The mean distance measurement error based solely on the frequency-estimation method is found to be approximately ±40 μm for the 60 GHz FMCW radar and ±30 μm for the 122 GHz FMCW radar.
- After the second CZT iteration, the bin width becomes extremely small (i.e., of the order of mHz) and therefore the undesirable effects present in a real scenario easily result in a slight frequency drift of the target peak. Consequently, a higher number of the CZT iterations could not lead to a further improvement of the distance measurement accuracy.
- The phase-estimation method improves the distance measurement accuracy of both the 60 GHz and 122 GHz FMCW radar by nearly a factor of 10. This tremendous improvement is attributed to the fact that IF signal phase is more sensitive to tiny changes in the target range (over an unambiguous range of $\lambda_{\mathrm{start}}/2$).
- The variance of the distance measurement error measured using the 122 GHz FMCW radar is nearly four times lower than the 60 GHz FMCW radar. Therefore, the CRLB of phase estimation for an FMCW radar has been experimentally validated. After combined frequency- and phase-estimation of 5100 continuous distance measurements, the accuracy of the 60 GHz FMCW radar was found to be 3.2 μm±11.4 μm and the accuracy of the 122 GHz FMCW radar was found to be 2.6 μm±5.7 μm (the accuracy is specified as mean error ±3σ for a normal distribution curve).

## Figures and Tables

**Table 1:** Parameters of the 60 GHz and 122 GHz FMCW radar.

Parameter	60 GHz FMCW Radar	122 GHz FMCW Radar
Start frequency ($f_{\mathrm{start}}^{\;}$)	57.5 GHz	120 GHz
Bandwidth (B)	5 GHz	5 GHz
Sweep time ($T_{\mathrm{sweep}}^{\;}$)	256 μs	384 μs
Tx power ($P_{\mathrm{Tx}}^{\;}$)	0 dBm	–3 dBm
Tx/Rx gain with lens (G)	25 dBi	25 dBi

**Table 2:** Measurement summary of the 60 GHz and 122 GHz FMCW radar.

	FFT + CZT	FFT + 2 × CZT	FFT + 2 × CZT + Phase	
	Mean Error	Mean Error	Mean Error	σ
**60 GHz FMCW Radar**	81 μm	42 μm	3.2 μm	3.8 μm
**122 GHz FMCW Radar**	40 μm	33 μm	2.6 μm	1.9 μm
